# Preparation of High Elongation and Low Hysteresis Conductive Hydrogels Strain Sensor Using Flake-like PEDOT Particles as Conductive Fillers

**DOI:** 10.3390/gels12060536

**Published:** 2026-06-15

**Authors:** Xiyuan Duan, Shimin Wang, Daheng Wang, Yu Gong, Ziwei Jiang

**Affiliations:** 1 School of Materials Science and Engineering, Hubei University, 368 Youyi Avenue, Wuchang District, Wuhan 430062, China; dxyce@live.com (X.D.); gy3212026@163.com (Y.G.); jzw0552@163.com (Z.J.); 2 School of Materials Science and Engineering, Wuhan University of Science and Technology, 947 Heping Avenue, Qingshan District, Wuhan 430081, China

**Keywords:** conductive hydrogel strain sensor, PEDOT particle, elongation, hysteresis, wearable electronic skin

## Abstract

Conductive hydrogel strain sensors using poly(3,4-ethylenedioxythiophene) (PEDOT) as fillers are rapidly advancing and are emerging as candidates for monitoring devices such as wearable electronic skin. However, due to limitations such as low elongation and high hysteresis, it is difficult to fully leverage its promising sensor properties in practical applications. In this study, we synthesized flake-like PEDOT particles (FP particles) and used Polyacrylamide (PAM) as the hydrogel matrix to fabricate a conductive hydrogel strain sensor. These particles were obtained by grinding PEDOT particles prepared via a template-free method. After swelling with ethylene glycol (EG) and assembly with polyvinyl alcohol (PVA), the FP particles become porous and contain many hydroxyl groups. This design enables the adsorption of acrylamide (AM) monomers within FP particles, facilitating the in situ polymerization of PAM onto the PEDOT/PVA chains, thereby yielding a dual-network structure with strong entanglements. This gives the sensor high elongation and very low hysteresis. In addition, it offers favorable sensor performance, including high sensitivity, high repeatability, and reliability. This strain sensor can be used in wearable electronic skin applications for facial monitoring and motion detection.

## 1. Introduction

Conductive hydrogel strain sensors are promising materials for constructing soft electronic sensors owing to their high-water content, high flexibility, and properties similar to those of human tissue [1,2,3,4]. To ensure accurate monitoring and long-term stable operation, next-generation strain sensors must offer high elongation, high sensitivity, low hysteresis, and reliable stability. Adding conductive fillers to a hydrogel matrix is the most common and cost-effective method for preparing conductive hydrogel strain sensors. Common conductive fillers include metal/metal oxide materials [5,6,7], carbon nanomaterials [8,9], composite fillers [10,11,12], and conductive polymers [13,14]. Among these, conductive polymers offer advantages such as high flexibility, high stability, and high conductivity.

Due to their high conductivity and water solubility, poly(3,4-ethylenedioxythiophene): polystyrene sulfonate (PEDOT: PSS) conductive inks are among the best choices for fabricating conductive hydrogel strain sensors [15,16,17]. However, due to the inherent limitations of rigid polymers, sensors made with PEDOT: PSS as a filler often exhibit low elongation, which limits their ability to function properly in environments with high strain. Many researchers have focused on improving the hydrogel matrix, such as by introducing a multi-network structure [17,18,19,20,21]. Prameswati et al. [22]. developed a sodium chloride-treated PEDOT: PSS/polyvinyl alcohol (PVA) conductive hydrogel. This conductive hydrogel exhibits high elongation (≈200%) and good elongation (150%) after healing. Li et al. [17] achieved a densified double-network hydrogel (5.5 wt.% conducting polymer) by concentrating a poorly crosslinked precursor hydrogel with a high conducting-polymer content. This hydrogel exhibits high electrical conductivity (≈10 S/cm) and a large fracture strain (≈150%). Soni et al. [23] prepared PEDOT: PSS/Polyacrylamide (PAM) hydrogels by varying the amount of N, N′-methylenebisacrylamide. The properties of these hydrogels can be tuned between high elongation/low strength and low elongation/high strength. Although these studies have made significant progress, the elongation and hysteresis of hydrogels remain less than ideal. While these methods can improve the sensor’s elongation, they often come at the expense of its hysteresis. Hysteresis is closely related to the sensor’s elasticity and fatigue resistance, all of which affect its long-term performance.

**Scheme 1:**
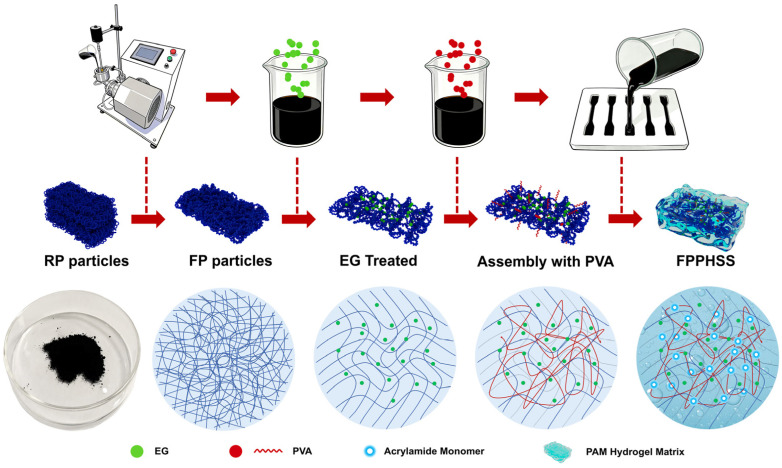
Schematic of the process and mechanism of preparation of Flake-like PEDOT/PAM Hydrogel Strain Sensor.

Many studies report that the network structure significantly influences hysteresis [24], elasticity [25], and fatigue resistance [26,27] of hydrogels. We believe that the spatial inhomogeneity of dual networks is a major cause of the decline in mechanical properties [28], and that the primary cause of this inhomogeneity may lie in PEDOT: PSS. Furthermore, the entanglement between these PEDOT: PSS and matrix networks is not very tight, leading to energy dissipation during stretching (such as the breaking of weak intermolecular forces between PEDOT and PVA, breaking of hydrogen bonds, disentanglement of long chains, and slippage), resulting in high hysteresis. Therefore, we propose a method for preparing hydrogel strain sensors using modified flake-like PEDOT particles (FP particles) as a conductive filler (Scheme 1). We synthesized PEDOT particles by a non-template method and ground them into flake-like particles using high-energy mechanical grinding. Subsequently, we modified them with ethylene glycol (EG) and assembled them with PVA. We selected PAM as the hydrogel matrix for the strain sensor.

FP particles used as fillers differ fundamentally from PEDOT: PSS. FP particles modified with EG and assembled with PVA can be regarded as a micro-conductive network. Because EG swells the FP particles, PVA molecules can permeate into them and assemble with PEDOT chains, further increasing the interchain spacing in PEDOT. Since the assembled FP particles contain a large number of PVA chains, the hydroxyl groups provided by these PVA chains can form hydrogen bonds with the amide groups of acrylamide (AM) monomer in the precursor. Therefore, during polymerization, AM forms PAM in situ on the PEDOT/PVA chains. Since FP particles are essentially composed of cross-linked and aggregated PEDOT molecular chains, the network itself is robust and difficult to disrupt. The interior provides numerous physical entanglement points, enabling the newly polymerized PAM chains to form strong entanglements with the PEDOT chains. This design significantly reduces uneven bonding between the dual networks within the system, thereby enhancing mechanical properties such as elongation. Furthermore, because the PEDOT chains are tightly entangled with the PAM, it is difficult for the two networks to become disentangled or slip relative to one another. When an external force is applied to the hydrogel, mechanical strain dissipates through entropic elasticity. Consequently, sensors prepared from these FP particles and PAM exhibit very low energy dissipation. Furthermore, since FP particles are prepared from PEDOT particles with high intrinsic conductivity, they serve as a natural conductive network with high conductivity and reliability. Consequently, the sensor’s performance should also remain good. Such stretchable sensors, which combine desirable mechanical properties with superior sensor performance, can deliver satisfactory performance in applications such as wearable electronic skin. Experiments demonstrate that the sensor, as a wearable electronic skin, can rapidly and accurately capture subtle changes in human facial expressions and exhibits excellent performance in large-scale human motion-capture scenarios.

## 2. Results and Discussion

### 2.1. Preparation of Flake-like PEDOT Particles

The non-template method is one of the earliest methods for synthesizing raw PEDOT particles (RP particles). Research indicates that the growth process of PEDOT particles synthesized via the non-template method proceeds from a linear structure to a coral-like structure, then to a flake-like structure, and finally to the aggregation of PEDOT particles [29,30]. Inspired by this approach, we believe that grinding of agglomerated particles can yield FP particles. Agglomerates of polymer nanoparticles are difficult to break up, so we opted for a high-energy mechanical grinding system. As a high-energy grinding machine, the horizontal grinder uses micron-sized zirconia beads as grinding media and can reach speeds over 3000 rpm, making it easy to break down agglomerated particles [31,32,33,34,35].

**Figure 1:**
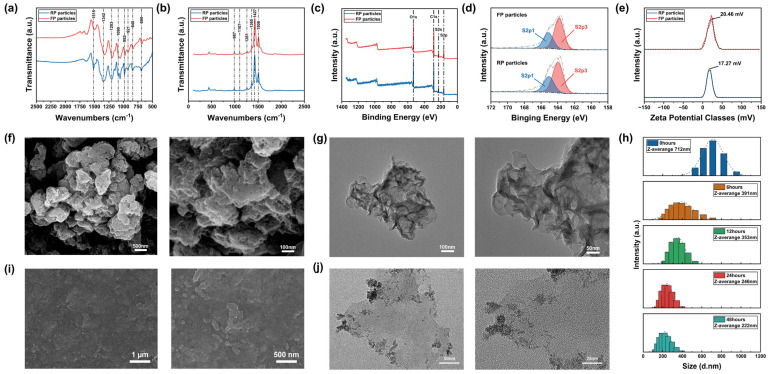
Characterization of PEDOT particles. (**a**) FTIR spectra of the RP and FP particles; (**b**) Raman spectra of the RP and FP particles; (**c**) XPS spectroscopy of the RP and FP particles; (**d**) The S 2p peak in the XPS spectroscopy of the RP and FP particles; (**e**) Zeta potential of the RP and FP particles; (**f**) SEM images of the RP particles; (**g**) TEM images of the RP particles; (**h**) DLS data of particles after different grinding times; (**i**) SEM images of the FP particles; (**j**) TEM images of the FP particles.

We first synthesized RP particles using a non-template method and characterized them. The FTIR spectra of RP particles are shown in Figure 1a. The peak at 1515 cm^−1^ corresponds to the C=C stretching vibration on the thiophene ring; the 1342 cm^−1^ peak corresponds to the C–C asymmetric stretching vibration on the thiophene ring; the 1203 cm^−1^, 1090 cm^−1^, and 982 cm^−1^ peaks correspond to the characteristic absorption peaks of the C–O–C bond of dioxythiophene, and the 921 cm^−1^, 840 cm^−1^, and 690 cm^−1^ peaks belong to the C–S–C bond bending vibration. The Raman spectrum of PEDOT is presented in Figure 1b. The following assignments are made: 987 cm^−1^ corresponds to oxyethylene ring deformation; 1107 cm^−1^ corresponds to C–O–C deformation; 1261 cm^−1^ corresponds to Cα–Cα′ (inter-ring) stretching; 1365 cm^−1^ corresponds to Cβ–Cβ′ stretching; 1427 cm^−1^ corresponds to symmetric Cα=Cβ stretching; and 1508 cm^−1^ corresponds to Asymmetric Cα=Cβ stretching. The elemental composition of the PEDOT particles was determined by XPS (Figure 1c,d). The peaks of O1s, C1s, S2s, and S2p are clearly observed in the figure. These data mentioned above are similar to those in other reports. The dispersion of the particles in water was characterized by Zeta potential (Figure 1e), and the results (17.27 mV) indicated moderate dispersion.

SEM images (Figure 1f) clearly reveal that the RP particles are composed of numerous small, flake-like blocks stacked together. At the edges of the particles, the irregularly stacked, misaligned morphology is clearly visible. TEM images of the RP particles (Figure 1g) clearly reveal distinct regions of varying shades across the entire particle. These areas of varying shades represent regions with differing degrees of stacking. Darker regions indicate relatively higher stacking of FP particles, while lighter regions indicate relatively lower stacking of FP particles.

We disperse the RP particles in water, stir thoroughly to ensure even distribution, then pour the dispersion into a horizontal grinder to begin grinding. Figure 1h shows changes in particle size over different grinding times. After 24 h of grinding, the average particle size was reduced to 246 nm, to 34.5% of its original size. Extending the grinding time to 48 h does not significantly alter the particle-size distribution and average particle size (222 nm). This indicates that after 24 h of processing, grinding efficiency declines as particles approach the limits of the horizontal grinder. We infer that, 24 h after grinding, most of the RP particles have been crushed into FP particles.

To determine whether the high-energy grinding process affects the chemical composition of the PEDOT particles, we dried the ground dispersion, isolated the dried FP particles, and characterized them. Their properties are almost consistent with those of RP particles. After grinding, the zeta potential showed a significant increase (20.46 mV), which was due to the fact that the RP particles were thoroughly crushed and their particle size significantly decreased. We diluted the dispersion (ground for 24 h), loaded it onto a silicon wafer, and dried it. In the SEM images of this sample (Figure 1i), no distinct RP particles were observed, and the FP particles were uniformly stacked on the silicon wafer to form a thin film. These SEM images provide indirect evidence that FP particles have been successfully synthesized. We examined the morphology of individual FP particles using TEM images (Figure 1j). Compared with the TEM image of the RP particles, fewer regions exhibit uneven light-and-dark patterns. The stacking of the FP particles has been significantly reduced, with only minor irregularities at the edges. These minor irregularities may be caused by trace amounts of smaller free particles adhering to the FP particles.

### 2.2. Design of the Flake-like PEDOT/PAM Hydrogel Strain Sensor

The modification of FP particles was based on studies of polyol-modified PEDOT: PSS films. The mechanism of polyol-modified PEDOT: PSS films is that the hydroxyl groups in the polyol form weak hydrogen bonds with the oxygen atoms in the PEDOT structural units, causing them to bind together (although some studies suggest this is due to polar interactions), swelling the PEDOT chains and causing the PSS to peel off [36,37,38,39].

Given polyols’ properties, we developed a method to modify FP particles with polyols and used it to fabricate conductive hydrogel strain sensors. EG is a commonly used modifier for PEDOT: PSS film modification. We used EG as a modifier to treat the FP particles. EG can permeate the FP particles and bind to the PEDOT chains, swelling the FP particles and loosening the PEDOT chains within them. Subsequently, we used PVA, another commonly used polyol modifier, to assemble the FP particles, following the same principle as before. Since PVA is a long-chain polymer, it further increases the distance between PEDOT chains. This creates more space, facilitating the permeation of AM. AM penetrates into the FP particles, and its amide groups form hydrogen bonds with the hydroxyl groups of PVA. Through this mechanism, PAM can polymerize in situ within the FP particles and form strong entanglements with the PEDOT chains. In a hydrogel, incompatible phases segregate, with fillers expelled from the matrix, forming isolated agglomerates [40,41,42]. Although there is no chemical cross-linking between PEDOT and PAM, sufficient physical cross-linking is sufficient to firmly embed the FP particles within the PAM. Furthermore, the PVA remaining within the FP particles acts as a bridge between the PEDOT and PAM chains, enhancing the compatibility between the two networks.

We prepared Flake-like PEDOT/PAM Hydrogel Strain Sensor (FPHS) using FP particle dispersions with varying concentrations of EG and PVA (Figure S1). It can be observed that, in the absence of any modifiers, FP particles are completely unable to interact with PAM. A distinct phase separation occurs, and the particles are completely expelled from the PAM. When only PVA was added, the FP particles still exhibited clear phase separation from the PAM, indicating that the PVA had not fully assembled with the FP particles. On this basis, as the amount of EG added increases, the phase separation between the FP particles and PAM is reduced, and the sensor gradually becomes more uniform. This indicates that FP particles must swell to some extent due to EG permeation, allowing PVA to enter, further loosening their structure and immobilizing the AM monomers. In addition, we investigated the case where only EG was added. Without PVA, significant phase separation still occurred. As the PVA concentration increased, phase separation gradually disappeared. This indicates that EG alone is insufficient to fully swell and loosen the FP particles, thereby accommodating more AM monomers. Consequently, the resulting PAM cannot fully entangle with the PEDOT chains. Furthermore, due to its low molecular weight, EG cannot act as a cross-linking agent to improve the compatibility between PEDOT and PAM.

**Figure 2:**
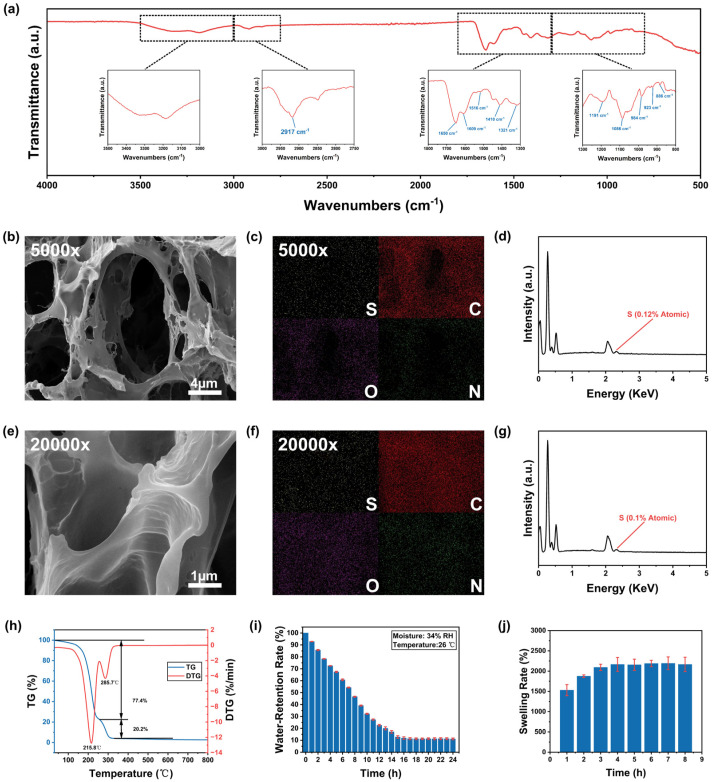
The characterization of FPHS. (**a**) FTIR spectra of the FPHS; (**b**) SEM image of the FPHS at 5000×; (**c**) Mapping of the FPHS at 5000×; (**d**) Elemental concentration of sulfur at 5000×; (**e**) SEM image of the FPHS at 20,000×; (**f**) Mapping of the FPHS at 20,000×; (**g**) Elemental concentration of sulfur at 20,000×; (**h**) TG/DTG curves of the FPHS; (**i**) Water-Retention Rate of the FPHS in 34%RH/26 °C; (**j**) Swelling Rate of FPHS in 26 °C.

FTIR spectra (Figure 2a) revealed the key components of the FPHS. The broad peak between 3000 cm^−1^ and 3600 cm^−1^ is due to the effects of the –OH stretching vibrations in EG and PVA, and the N–H stretching vibrations in PAM. The peak near 2917 cm^−1^ corresponds to C–H stretching vibrations from aliphatic hydrocarbons. The peak near 1650 cm^−1^ corresponds to the amide C=O stretching vibrations in PAM and MBA. The peak near 1609 cm^−1^ corresponds to the N–H bending vibration. The peak near 1516 cm^−1^ corresponds to C=C stretching vibrations from PEDOT. The peak near 1410 cm^−1^ corresponds to C–N stretching vibrations from PAM. The peak near 1321 cm^−1^ corresponds to C–C asymmetric stretching vibration on the thiophene ring. The 1191 cm^−1^, 1086 cm^−1^, and 984 cm^−1^ peaks correspond to the characteristic absorption peaks of the C–O–C bond of dioxythiophene, and the 923 cm^−1^ and 886 cm^−1^ peaks belong to the C–S–C bond bending vibration. Analysis of the FTIR spectrum confirmed that FPHS had been successfully synthesized.

To verify the dispersion of FP particles in PAM, we obtained SEM images and EDS data of the FPHS and analyzed the elemental composition and distribution at different magnifications. Figure 2b,e presents SEM images of the FPHS at different magnifications (5000× and 20,000×). In the image, the surface of the hydrogel pores’ walls is smooth, and no FP particles are clearly visible. We believe this indicates that the FP particles have been encapsulated within the PAM. Figure 2c, Figure 2d, Figure 2f, and Figure 2g show the elemental distribution and concentration data from EDS at 5000× and 20,000×, respectively. Since sulfur is a characteristic element of the FP particles in the sensor, the sulfur content reflects their distribution within the sensor. At both magnifications, the distribution of sulfur is uniform, with no obvious localized accumulation. In addition, the sulfur content is essentially the same at both magnifications. This indicates that the distribution of FP particles in PAM is notably uniform.

For comparison, Figure S2a shows SEM and EDS data for a sensor prepared using the same method, with the only change being the replacement of FP dispersion with RP dispersion. As shown, distinct RP particles are visible on the hydrogel walls. Furthermore, the concentration of sulfur (S) is higher and more concentrated at the locations of these RP particles.

In addition, to verify the reproducibility of the preparation method, Figure S2b shows SEM and EDS data for different batches and locations under the same process conditions. These data demonstrate high consistency, indicating that FP particles can be stably and uniformly embedded in PAM. This demonstrates that the preparation method for FPHS is highly reproducible and stable.

We conducted a study on the stability of FPHS to ensure its suitability for practical applications. Figure 2h shows the TG/DTG curves for FPHS. As the temperature rises, the water in FPHS evaporates first. Since ethylene glycol (EG) has a boiling point of approximately 197 °C, its evaporation overlaps with that of water in the 100–250 °C range, together forming a distinct weight-loss region that reaches a maximum rate near 215.8 °C. Subsequently, other components in FPHS (including FP particles, PVA, and PAM) begin to decompose. The thermal decomposition temperatures of these substances are relatively close, and a second peak in the rate of weight loss appears near 285.7 °C. Figure 2i shows the change in water retention of FPHS over time at 26 °C and 34% relative humidity (FPHS had reached swelling equilibrium prior to testing). As shown in the figure, the water retention of FPHS shows a clear downward trend over time, with a phased character. During the first 10 h, the water retention rate dropped sharply, from 100% to approximately 32%. Subsequently, the rate of water loss gradually slowed and stabilized after 16 h (entering a plateau phase). After 24 h of testing, FPHS ultimately maintained a water retention rate of approximately 11%. Figure S3 illustrates the evolution of the water retention rate of FPHS over time under severe test conditions (45 °C, humidity < 10% RH). As shown in the figure, even in a dry, high-temperature environment, FPHS still retains some water in the early stages. After 1 h in a high-temperature, low-humidity environment, the material retained approximately 60% of its initial water content; after 2 h, it dropped to approximately 20%, and after 3 h, it was about 10%. As time progressed, the rate of water loss gradually slowed and stabilized; by the end of the 12 h test, the material retained approximately 4% of its final residual water. These data demonstrate that FPHS exhibits good water retention and meets the requirements of practical applications. Figure 2j illustrates the change in the swelling ratio of FPHS over time. As shown in the figure, this FPHS exhibits exceptional water absorption performance. During the initial water absorption phase (within 1–4 h), the material exhibited rapid swelling, with the swelling rate surging to approximately 1500% within 1 h and stabilizing at swelling equilibrium (approximately 2200%) within 4 h. This indicates that FPHS not only has a high swelling capacity but also exhibits rapid water absorption.

### 2.3. Mechanical Properties

**Figure 3:**
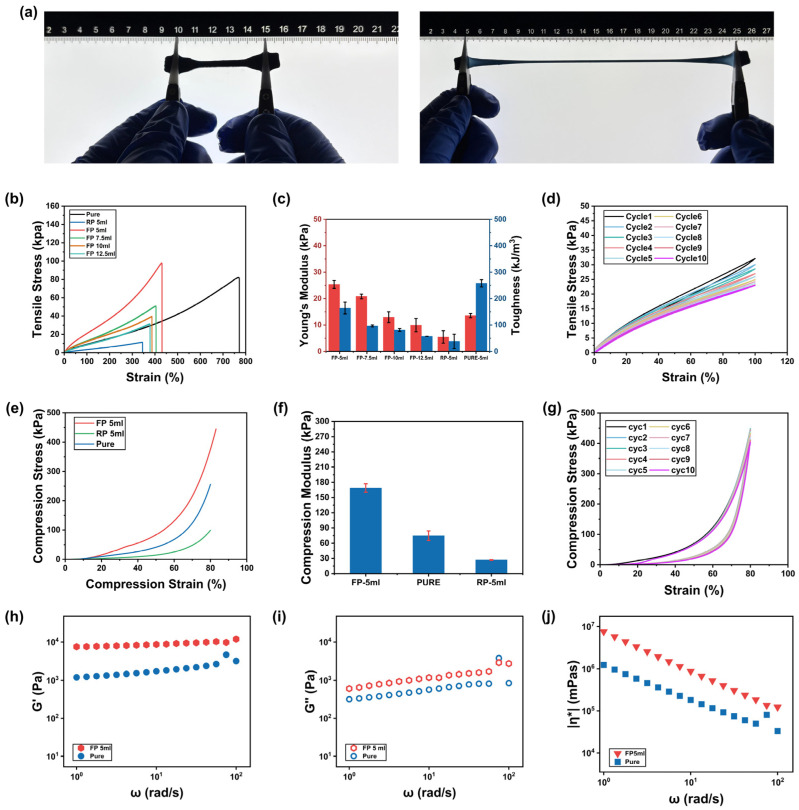
Mechanical properties of the FPHS. (**a**) Photographs of the as-prepared and stretched FPHS; (**b**) Tensile stress−strain curves of different hydrogels (Maximum value in parallel samples); (**c**) Young’s modulus and toughness of different hydrogels; (**d**) Loading/unloading curves of the FPHS (FP 5 mL) at 100% strain for 10 consecutive times; (**e**) Compressive stress−strain curves of different hydrogels; (**f**) Compression modulus of different hydrogels; (**g**) Compressive-recover curves of the FPHS (FP 5 mL) with the maximum compress strain of 80%; (**h**) Storage modulus (G′) of FPHS (FP 5 mL) and Pure hydrogel; (**i**) Loss modulus (G″) of FPHS (FP 5 mL) and Pure hydrogel; (**j**) Complex viscosity ($|\eta^{*}|$) of FPHS (FP 5 mL) and Pure hydrogel.

Because FP particles form strong entanglements with PAM, the mechanical properties of the FPHS exhibit unique characteristics. FPHS has High Elongation (Figure 3a). The maximum tensile strain of FPHS can reach up to a 431.23% (Figure 3b, FP-5 mL) while the maximum tensile stress reached 97.78 kPa. The maximum tensile strain is significantly higher than that of pure PEDOT: PSS hydrogels and most other PEDOT: PSS composite hydrogels (Table 1). In particular, compared to PEDOT: PSS hydrogels that use PAM as the matrix, FPHS can simultaneously achieve both the high tensile strain and the high tensile stress [23]. In addition, compared to pure PAM hydrogels, FPHS exhibits a higher Young’s modulus (Figure 3c), indicating that the incorporation of FP particles significantly enhances strength. This property—which combines high elongation with enhanced strength—highlights the advantages of FP particles as a conductive component.

To investigate the hysteresis of the FPHS (FP-5 mL), we subjected it to loading/unloading cycling tests at 100% strain (Figure 3d). A small hysteresis loop was observed in the first cycle, primarily due to the initial relaxation of the hydrogel’s network and the breaking of hydrogen bonds between trace amounts of PVA, resulting in residual strains that could not be fully recovered. In subsequent cycles, the hysteresis loop rapidly decreased, and by the third cycle, it was virtually absent. After the eighth cycle, the curves overlap almost completely (DH = 2.18%), and the maximum tensile stress remains virtually unchanged. This low hysteresis is almost absent in other PEDOT: PSS conductive hydrogels. We attribute this pronounced difference to the degree of entanglement between the PEDOT and PAM chains. Generally, the more entanglement points there are between hydrogel molecular chains, the more difficult it is to untangle them, which results in a narrower hysteresis loop and a smaller area. In most hydrogels prepared by directly adding PEDOT: PSS, the PEDOT chains exist as free species in the precursor solution and tend to adopt a compact, coiled conformation due to the exclusion effect of the hydrogel matrix. Under these conditions, the PEDOT chains have difficulty forming tight entanglements with the hydrogel matrix chains. During stretching, slippage and disentanglement readily occur between the PEDOT chains and the matrix, resulting in pronounced hysteresis. Moreover, PEDOT: PSS contains a large amount of PSS, which cannot form strong interactions with the hydrogel matrix. As the hydrogel is repeatedly stretched, these PSS chains also undergo molecular slippage relative to the hydrogel matrix. In contrast, in the FPHS, the PEDOT chains are first modified and swollen with EG and PVA, forming a loose, flake-like structure. AM monomers then permeate this structure and polymerize in situ, generating PAM chains that become entangled with the PEDOT chains. These entanglements behave like permanent knots that are extremely difficult to disentangle. During tensile loading, mechanical strain is dissipated through viscoelasticity. As a result, the hysteresis exhibited by the FPHS is far lower than that of hydrogels using PEDOT: PSS as filler.

We also found that, depending on the amount of FP dispersion used, changes in tensile properties exhibit a consistent pattern. As the amount of FP dispersion increases, the maximum tensile strain, Young’s modulus, and toughness all decrease. When 5 mL of FP dispersion was used, the maximum tensile strain, Young’s modulus, and toughness reached their peak values. This pattern has also been reported in similar studies of PEDOT: PSS hydrogels [22,45]. We believe that because the PAM matrix cannot fully accommodate these FP particles, excessive addition will inevitably lead to FP particle agglomeration. Agglomerated FP particles will cause significant defects, resulting in a sharp decline in tensile properties. In addition, we prepared hydrogels using RP particles via the same method. The results showed that their mechanical properties were notably poor, rendering them virtually unusable. This is because the RP particles are coarse and tightly packed, making it difficult for EG to penetrate sufficiently, thereby preventing the assembly of PVA and the penetration of AM. These coarse particles are incompatible with PAM, leading to severe phase separation and significant mechanical defects at their sites.

The compressive properties of FPHS have also been studied. Figure 3e shows the stress–strain curves at 80% strain. At 80% strain, compression stress increases sharply. Among these, the FPHS (FP-5 mL) achieved a maximum compression stress of 445 kPa, while the RP hydrogel and the Pure PAM hydrogel reached 99 kPa and 256 kPa, respectively. As shown in Figure 3f, the compression modulus of FPHS is significantly higher than that of the pure PAM hydrogel. This reflects a significant enhancement in the FPHS’s compressive strength, driven by the incorporation of FP particles, which increases overall structural strength. Additionally, the FPHS can recover 10 times at 80% compressive strain, indicating favorable recovery of the hydrogel network (Figure 3g).

To investigate whether AM monomer molecules penetrated into the FP particles and underwent in situ polymerization to form PAM, thereby creating a structure with tightly entangled molecular chains, we performed dynamic oscillatory shear measurements in frequency sweep mode. The storage modulus (G′) reflects the material’s capacity for elastic deformation and is closely related to the density of physical crosslinking points or chain entanglements within the hydrogel. As shown in Figure 3h, the G′ values of FPHS are significantly higher than those of the Pure hydrogel over the entire tested frequency range. In the low-frequency region, G′ of the former is approximately 8 × 10^3^ Pa, whereas that of the latter is around 1 × 10^3^ Pa—a difference of nearly one order of magnitude. This indicates that a denser three-dimensional network structure forms within FPHS, and the degree of physical entanglement between molecular chains is far greater than in the Pure hydrogel. Figure 3i presents the loss modulus (G″) as a function of angular frequency. Similarly, the G″ values of FPHS are higher than those of the Pure hydrogel. Importantly, a comparison of Figure 3h,i reveals that for FPHS, G′ is much larger than G″, displaying typical elasticity-dominated behavior, whereas for the Pure hydrogel, the difference between the two moduli is relatively smaller. This pronounced G′ ≫ G″ characteristic further confirms the stronger molecular chain confinement and the high-density entanglement network within FPHS. A comparison of the complex viscosity (|η*|) provides direct support for the above conclusions. As shown in Figure 3j, the complex viscosity of FPHS is significantly higher than that of the Pure hydrogel across the entire frequency range. In particular, at a low shear frequency (10^0^ rad/s), its viscosity reaches nearly 10^7^ mPas, which is far higher than the 10^6^ mPas recorded for the Pure hydrogel. The high viscosity and the pronounced shear-thinning behavior imply intense intermolecular friction and steric hindrance effects, which are direct manifestations of strong physical entanglements among long polymer chains. The dynamic rheological analysis consistently demonstrates that the internal network of the FPHS hydrogel exhibits denser and more robust molecular chain entanglements (i.e., effective crosslinking points) than that of the Pure hydrogel, resulting in a significant increase in both the elastic modulus and the steady-state viscosity. This indicates that the AM monomers permeated the particles and polymerized in situ to form PAM, which then became tightly entangled with the PEDOT chains.

These results indicate that when FP particles are used as a conductive network, they can significantly enhance the mechanical properties of the hydrogel. The robust mechanical properties demonstrated by FP-P hydrogel support its potential applications in wearable monitoring devices.

### 2.4. Sensor Properties

FPHS combines high elongation with low hysteresis, making it a promising material for sensor applications. At the same time, thanks to the robust conductive network formed by FP particles within the PAM, it also exhibits good sensor performance.

**Figure 4:**
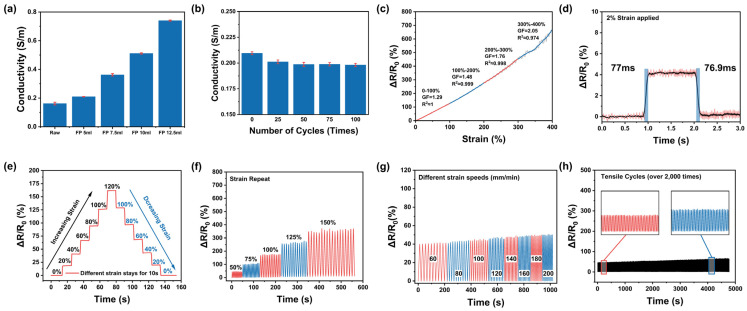
Sensing properties of the FPHS. (**a**) Static electrical conductivity of FPHS with different FP particle contents; (**b**) static conductivity after 25, 50, 75, and 100 tensile cycles at 100% strain; (**c**) the gauge factor of the FPHS changes with strain; (**d**) the response time of the FPHS at 2% strain applied; (**e**) resistance response of the FPHS at different strains for 10 s; (**f**) resistance response of the FPHS cycled at 50%, 75%, 100%, 125%, and 150% strain; (**g**) resistance response of the FPHS cycled under different tensile rates; (**h**) resistance response of the FPHS cycled over 2000 times at 50% strain.

The intrinsic conductivity of PEDOT confers electrical conductivity to the FPHS. We systematically evaluated the static electrical conductivity of FPHS as a function of FP particle content (Figure 4a). As the FP particle content increased, the electrical conductivity of the FPHS also gradually increased. Since sensor performance is not directly determined by the magnitude of electrical conductivity but rather by how conductivity changes under different conditions, and considering the mechanical properties, we selected the FPHS prepared with FP-5 mL for further study. We further analyzed the conductivity stability of this FPHS after 25, 50, 75, and 100 tensile cycles at 100% strain (Figure 4b). The results showed that after 25 cycles, there was a very slight decrease in conductivity; however, upon continued tensile cycling, the conductivity no longer changed significantly. This demonstrates the good stability of the FPHS’s conductivity under tensile stress.

The response of a strain sensor is characterized by the relative change in resistance, denoted ΔR/R_0_, where R_0_ is the initial resistance, and ΔR is the change in resistance relative to R_0_. The sensitivity of a sensor is typically quantified by gauge factor (GF), defined as the ratio of the relative change in resistance to the applied strain. The data shown in Figure 4c indicate that the FPHS’s response curve shows a clear upward trend with increasing strain. Significant changes in GF were observed at different stages of tensile testing. In the initial stage (0–100% strain), GF was 1.29, whereas during subsequent tensile, GF increased to 2.05 (300–400% strain). This indicates that the FPHS’s sensitivity increases significantly with increasing strain. In the low-strain regime, the conductive network remains well-connected, resulting in small changes in contact resistance, a gradual increase in resistance, and a relatively low gauge factor (GF). As the strain increases, the overlap between the conductive networks formed by FP particles decreases, and the contact points slip, causing a sharp rise in contact resistance. When the strain becomes sufficiently large, a substantial portion of the conductive networks becomes completely separated, leading to a nonlinear surge in resistance and a corresponding significant increase in GF. The GF curve indicates that its favorable sensitivity makes it suitable for most sensor applications. Figure 4d illustrates the response time of the FPHS sensor to tensile strain stimulation. When current signals are acquired at high frequency, the response time is approximately 77 ms (0–2% strain). This rapid response facilitates the detection of various subtle facial expression signals and intense human movement signals. In addition, the FPHS exhibited good stability and recovery under strain gradients ranging from 0% to 120% (Figure 4e). At the same strain level, the ΔR/R_0_ was nearly identical during both the tensile and recovery phases.

In different tensile-strain experiments (Figure 4f), the sensing response of the FPHS increased with increasing tensile strain. At the same time, the FPHS demonstrated good repeatability and recoverability during repeated tensile-recovery cycles. In addition, we investigated the response of the FPHS at different tensile rates (Figure 4g). The results show that its ΔR/R_0_ increases slightly with increasing tensile rate. In practical applications, the tensile rate of the target being monitored is not constant, indicating that the FPHS exhibits good adaptability to real-world monitoring scenarios. Figure 4h shows the results of cyclic tensile-recovery testing of the FPHS, which was repeated over 2000 times at a strain of 50%. During cycling, ΔR/R_0_ showed a slight upward trend. During cycling, ΔR/R_0_ showed a slight upward trend. This is primarily attributed to the inevitable internal damage to the sensor at high cycle counts. For instance, the FP particles could not fully return to their original positions, and some entangled molecular chains were broken or disentangled. Such damage is generally irreversible, indicating that the sensor has a finite service life in terms of cycling. Moreover, due to the test’s long duration, substantial water loss occurred, and continued stretching under these conditions accelerated sensor damage. However, this change remained at around 10% even after 2000 cycles, indicating that the FPHS possesses favorable durability and longevity.

### 2.5. Applications of Wearable Electronic Skin for Facial Monitoring and Human Motion Detection

**Figure 5:**
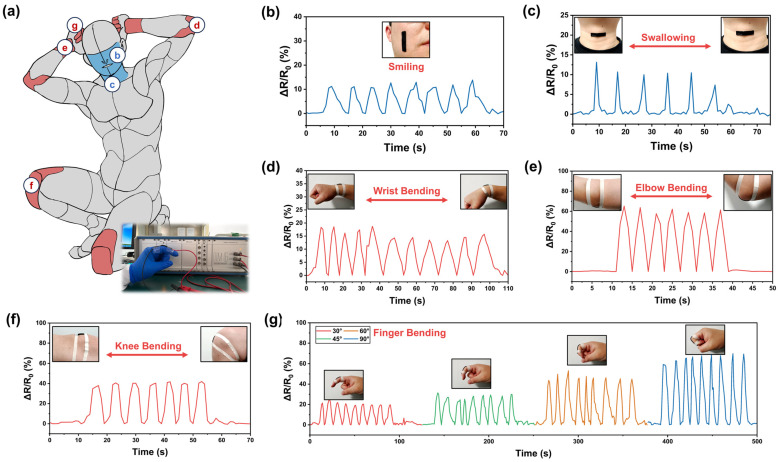
Facial monitoring and human motion detection. (**a**) The applications of FPHS to fabricate a wearable electronic skin for monitoring different parts of the human body and photo of actual monitoring; (**b**) work as a facial monitoring to capture the human smile; (**c**) work as a facial monitoring to capture the human swallow; (**d**) work in human motion detection to monitor the wrist bend; (**e**) work in human motion detection to monitor the elbow bend; (**f**) work in human motion detection to monitor the knee bend; (**g**) work in human motion detection to monitor the finger bend at different angles.

We used FPHS to fabricate a wearable electronic skin. Thanks to its simple fabrication process, we can quickly and easily produce large quantities of wearable electronic skin and use different molds to customize its shape to fit various body parts (Figure 5a). FPHS is encapsulated using PDMS to ensure stable performance and safe use (Figure S4).

Experimental results show that the FPHS wearable electronic skin, as a facial monitoring, can accurately capture subtle changes in the human face, including smiling and swallowing (Figure 5b,c). When a volunteer smiles, the sensor accurately captures facial changes and converts them into electrical signals. When the face returns to a neutral expression, the resistance stabilizes at its initial value. Similarly, the FPHS wearable electronic skin used on the throat can accurately detect and transmit subtle changes in the throat muscles during voluntary swallowing. These good performance characteristics are attributable to the FPHS’s high sensitivity, which enables it to provide clear feedback even in response to minute strains. This indicates that the FPHS wearable electronic skin can be used in a variety of applications, such as communication for patients with paralysis.

In terms of human motion detection, the FPHS wearable electronic skin also performs admirably. When applied to larger human joints, such as the wrist (Figure 5d), elbow (Figure 5e), and knee (Figure 5f), the FPHS also provides accurate feedback on their motion signals. Experimental results demonstrate that the FPHS wearable electronic skin is highly sensitive to joint motion signals. This indicates its suitability for applications such as precise human motion capture. In addition, we tested the reproducibility and reliability of the FPHS wearable electronic skin. Figure 5g shows the step-response of the MS hydrogel to the bending of a finger joint. The results show that the rate of change in resistance exhibits good repeatability and stability across different angles.

## 3. Conclusions

We describe the design and fabrication process of the flake-like PEDOT/PAM hydrogel strain sensor and characterize its mechanical and sensing properties. The results indicate that our sensors exhibit high elongation (430%) and low hysteresis (DH = 2.18%) when compared with others. This is because the PEDOT chains in FP particles, prepared via high-energy mechanical grinding, EG modification, and PVA assembly, can form strong entanglements with the PAM. We also tested the sensor performance, and the results demonstrated its advantages in terms of fast response (approximately 77 ms at 0–2% strain), high repeatability (at different tensile rates and strains), and high reliability (exhibits relatively stable repeatability over 2000 cycles). Based on these properties, we have developed a wearable electronic skin. It is suitable for applications such as precise facial monitoring and motion detection. This study not only addresses key challenges in developing high-performance strain sensors based on PEDOT conductive polymer hydrogels, but also offers new insights into selecting PEDOT conductive fillers.

## 4. Materials and Methods

### 4.1. Materials

3,4-Ethylenedioxythiophene (EDOT), Macklin Biochemical Technology Corporation (Shanghai, China); Ferric chloride hexahydrate, Aladdin Industrial Corporation (Shanghai, China); Anhydrous ethanol, Sinopharm Chemical Reagent Corporation; Acrylamide (AM), Aladdin Industrial Corporation (Shanghai, China); N′, N′-Methylenebis (MBA), Aladdin Industrial Corporation (Shanghai, China); Ammonium persulfate (APS), Aladdin Industrial Corporation (Shanghai, China); N, N, N, N-tetramethylethylenediamine (TEMED), Aladdin Industrial Corporation (Shanghai, China); Ethylene glycol (EG), Aladdin Industrial Corporation (Shanghai, China); Poly (vinyl alcohol), Macklin Biochemical Technology Corporation (Shanghai, China); distilled water, prepared in the Laboratory.

### 4.2. The Synthesis of PEDOT Particles

Take a beaker, add 150 mL of distilled water, then add 5 g of EDOT. Place the beaker on a low-temperature magnetic stirrer and reduce the temperature to 10 °C. Stir the EDOT aqueous dispersion for 30 min until fully dispersed. Weigh 23.77 g of ferric chloride and dissolve it in distilled water to prepare a 100 g solution of ferric chloride in water. Maintain low-temperature stirring while adding the ferric chloride solution to the beaker. After the addition is complete, raise the temperature to 50 °C and continue the reaction for 72 h. After the reaction is complete, use a sand core funnel to separate the PEDOT particles from the reaction solution. Rinse repeatedly with ethanol and water, then place the washed PEDOT particles in a vacuum drying oven and dry at 80 °C for 48 h.

### 4.3. The Process of Mechanical Grinding, EG Treatment, and Assembly with PVA

Flake-like PEDOT particle dispersion with varying solid content can be prepared by adjusting the mass ratio of Raw PEDOT particles to water. Typically, the solid content of Raw PEDOT particles should not be excessively high to prevent inadequate grinding, nor should it be too low to avoid excessive wear and damage to the grinding media. In this manuscript, a 2% wt concentration was used. Take 4 g of Raw PEDOT particles and add them to a beaker containing 100 mL of distilled water. Stir thoroughly to disperse it in the water. Pour 100 mL of distilled water into the horizontal grinder’s feed chamber. Turn on the grinder and set the speed to 3000 rpm to initiate water circulation. Slowly pour the aqueous dispersion of Raw PEDOT particles into the grinding hopper while stirring continuously. After grinding for the specified period, collect the resulting dispersion of Flake-like PEDOT particles.

Depending on the experimental system, appropriate amounts of ethylene glycol (EG), polyvinyl alcohol (PVA), FP particle dispersion, and water were mixed to form a 25 g mixture. The mixture was stirred at 90 °C for one hour to ensure complete dissolution of PVA. Finally, a dispersion of assembled sheet-like PEDOT particles was obtained. It should be noted that in experiments with different amounts of FP particle dispersion, a larger mass of the dispersion was compensated by a correspondingly smaller mass of distilled water. In this way, the total mass of the mixture was always maintained at 25 g, yielding mixtures that differed only in FP particle content for comparative tests. The same procedure was followed for experiments with varying EG and PVA contents.

### 4.4. The Synthesis of Flake-like PEDOT/PAM Hydrogel Strain Sensor

Transfer 5 mL of the dispersion of assembled Flake-like PEDOT particles to a beaker. Weigh 2 g of AM powder, 2 mL of MBA solution (4 mg/mL), and 3 mL of distilled water into the beaker. Stir thoroughly for 30 min to ensure all components are uniformly mixed. Maintain stirring while adding 40 mg of APS, and continue stirring for 10 min. Add 10 μL of TEMED while stirring. After stirring for 1 min, rapidly transfer the mixture to a mold. Place the mold in a 60 °C environment and maintain the temperature for 60 min to obtain the Flake-like PEDOT/PAM hydrogel strain sensors.

### 4.5. Characterization

Fourier-transform infrared (FT-IR) spectra of the PEDOT were obtained using a Nicolet iS50 spectrometer (Thermo Fisher Scientific, Waltham, MA, USA). Raman scattering data were collected using a DXR2xi (Thermo Fisher Scientific, Waltham, MA, USA). X-ray photoelectron spectroscopy was performed on the PEDOT particles using an ESCALAB 250Xi (Thermo Fisher Scientific, Waltham, MA, USA). Dynamic Light Scattering (DLS) data and Zeta potential data were collected by a Zetasizer Nano ZS90 (Malvern Panalytical, Malvern, UK). Scanning electron microscopy (SEM) data were collected using a TESCAN MIRA LMS (TESCAN, Brno, Czech Republic). Transmission electron microscope (TEM) data were obtained on FEI Talos F200X G2 (FEI, Hillsboro, OR, USA) and FEI Talos F200S (FEI, Hillsboro, OR, USA). Mechanical property data were obtained on a Shimadzu AGS-X-50N (Shimadzu, Kyoto, Japan). Electrical and sensing properties data were collected using a Zennium IM6 Electrochemical Workstation (Zahner-elektrik, Kronach, Germany). Dynamic oscillatory shear measurements were performed using a Haake Mars 40 (Thermo Fisher Scientific, Karlsruhe, Germany). TG/DTG data obtained using the Netzsch STA 2500 Regulus (NETZSCH, Selb, Germany).

### 4.6. Details of Mechanical and Sensor Properties Testing

The specimens were dumbbell-shaped with overall dimensions of 50.0 ± 0.5 mm (length) × 8.5 ± 0.5 mm (width) × 2.0 ± 0.2 mm (thickness). The narrow central gauge section measured 16 ± 1.0 mm in length and 4.0 ± 0.1 mm in width. In the tensile test, the loading rate was 20 mm/min, and three parallel tests were conducted. In the compression test, the loading rate was 0.5 mm/min, and three parallel tests were conducted.

In the dynamic oscillatory shear test conducted in frequency-sweep mode, the samples were circular, with a diameter of 20 mm ± 0.5 mm and a thickness of 2 mm ± 0.1 mm. The test was performed at a room temperature of 25 °C.

Sensor testing was performed using an electrochemical workstation set to a constant voltage of 1.5 V. The test results were recorded as voltage-current data, which was then converted to resistance values through calculation. Static conductivity was measured directly using a four-point probe method.

## Figures and Tables

**Table 1:** Comparison of tensile range and conductivity of this work with other reported hydrogel sensors using PEDOT: PSS as conductive network.

Materials	Maximum Tensile Strain	Hysteresis	Reference
pure	35%	-	[43]
PEDOT: PSS/PVA	300%	-	[44]
PEDOT: PSS/PVA	150%	large	[17]
PEDOT: PSS/PVA	<200%	large	[22]
PEDOT: PSS/PEGDA/PEG	900%	large	[18]
PEDOT: PSS/SA/PVA	413.65%	large	[19]
PEDOT: PSS/PAM	300~400% (low stress)	tiny (DH = 2.27%)	[23]
FP-Particles/PAM	430%	tiny (DH = 2.18%)	Our work

## Data Availability

The data that support the findings of this study are available from the corresponding authors upon reasonable request.

## Supplementary Materials

The following supporting information can be downloaded at: https://www.mdpi.com/article/10.3390/gels12060536/s1. Figure S1: Flake-like PEDOT/PAM hydrogel strain sensors made by different amounts of EG and PVA; Figure S2: Additional SEM and EDS data of hydrogels. (a) SEM and EDS data for hydrogels prepared by RP dispersion; (b) SEM and EDS data for different batches and locations of another flake-like PEDOT/PAM hydrogel. Figure S3: Water-Retention Rate of the FPHS in <10%RH/45 °C. Figure S4: FPHS Encapsulation Methods.

## Abbreviations

PEDOT	poly(3,4-ethylenedioxythiophene)
PEDOT: PSS	poly(3,4-ethylenedioxythiophene): polystyrene sulfonate
RP particles	raw PEDOT particles
FP particles	flake-like PEDOT particles
PVA	polyvinyl alcohol
AM	acrylamide
PAM	Polyacrylamide
EG	ethylene glycol
FPHS	Flake-like PEDOT/PAM Hydrogel Strain Sensor
