# Supplementary material for: Preparation of High Elongation and Low Hysteresis Conductive Hydrogels Strain Sensor Using Flake-like PEDOT Particles as Conductive Fillers
Source: Gels. 2026 Jun 15;12(6):536. doi: 10.3390/gels12060536 (PMC13299653; doi:10.3390/gels12060536)
Supplement: Supplementary file 1 [file gels-12-00536-s001.zip › gels-4338747-supplementary.pdf]

Support Information

Additional data

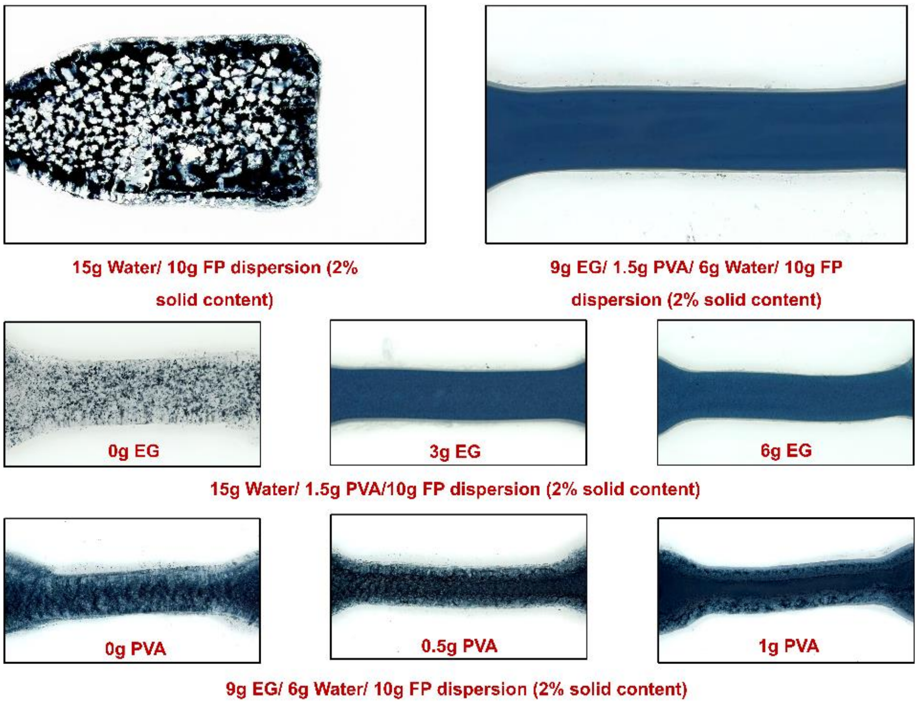

Figure S1. Flake-like PEDOT/PAM hydrogel strain sensors made by different amounts of EG and PVA

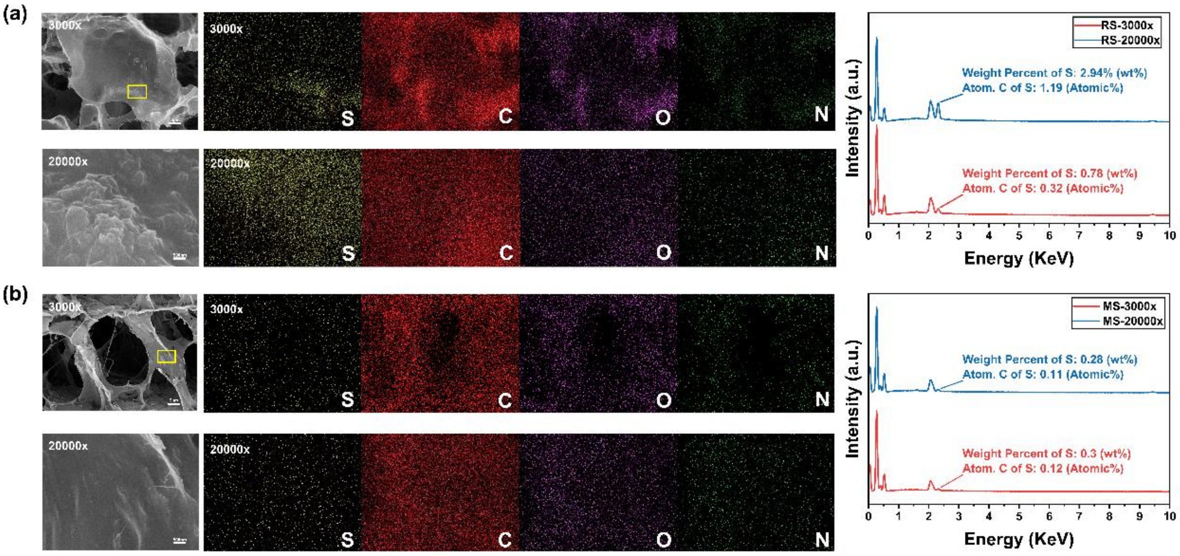

Figure S2. Additional SEM and EDS data of hydrogels. a) SEM and EDS data for hydrogels prepared by RP dispersion; b) SEM and EDS data for different batches and locations of another flake-like PEDOT/PAM hydrogel.

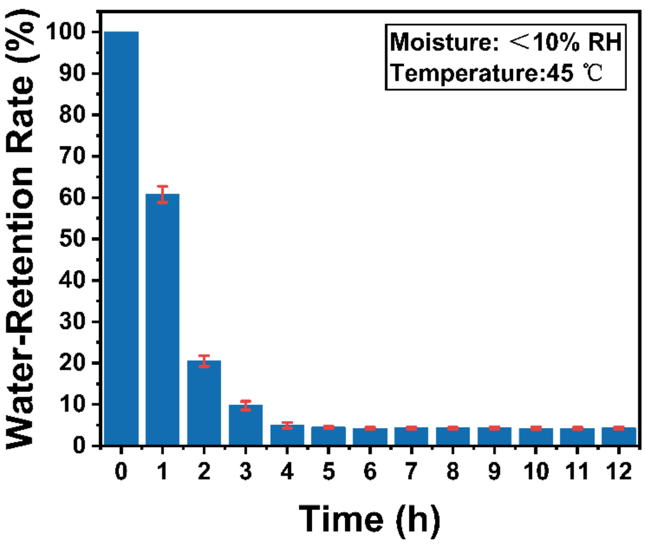

Figure S3. Water-Retention Rate of the FPHS in ＜10%RH/ 45°C.

Testing Guidelines for Wearable Applications

All FPHS units are encapsulated in PDMS film prior to use to prevent moisture loss and protect them from external influences.

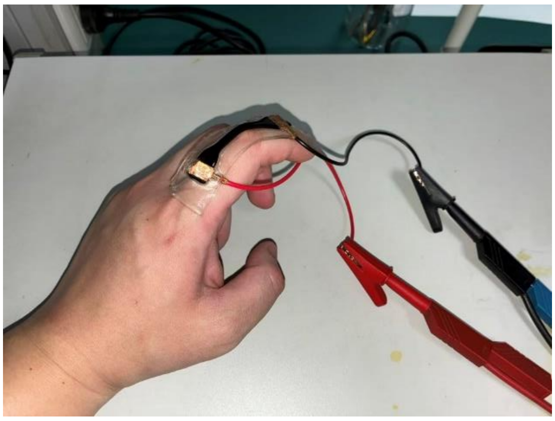

Figure S4. FPHS Encapsulation Methods.

Additionally, this encapsulation ensures safety for the user's skin, as no biocompatibility studies were conducted in this manuscript.
